# Antibiotic susceptibility and antagonistic activity of candidate probiotic strains relevant to poultry production: An in vitro screening study

**DOI:** 10.1016/j.onehlt.2026.101629

**Published:** 2026-10-24

**Authors:** Niloofar Akhavan, Katarzyna Stadnicka, Dominika Thiem, Gillian E. Gardiner, Peadar G. Lawlor, Cinzia Lucia Randazzo, Katarzyna Hrynkiewicz

**Affiliations:** a Department of Microbiology, Faculty of Biological and Veterinary Sciences, Nicolaus Copernicus University, Lwowska 1, 87-100 Torun, Poland; b Faculty of Health Sciences, Nicolaus Copernicus University in Toruń, Ludwik Rydygier Collegium Medicum, Łukasiewicza 1, 85-821 Bydgoszcz, Poland; c Institute for Advanced Studies, Wileńska 4, 87-100 Toruń, Nicolaus Copernicus University, Poland; d Eco-Innovation Research Centre, Department of Science, South East Technological University, Cork Road, Waterford X91K0EK, Co. Waterford, Ireland; e Teagasc, Pig and Poultry Research and Knowledge Transfer Department, Animal and Grassland Research and Innovation Centre, Moorepark, Fermoy, P61 C996 Co. Cork, Ireland; f Department of Agriculture, Food and Environment, University of Catania, Via Santa Sofia, Catania, Italy

**Keywords:** Probiotic safety, Poultry-associated, Foodborne pathogen, Antibiotic susceptibility, Candidate prioritization, Antagonistic activity, One health

## Abstract

The global pressure to reduce antibiotic use in poultry production, driven by antimicrobial resistance (AMR), highlights the need for safe and effective microbiota-based alternatives. This study performed an in vitro screening of 10 candidate probiotic strains (*Lactobacillus* spp., *Bifidobacterium lactis*, *Carnobacterium divergens*, and *Bacillus* spp.) to assess (i) antibiotic susceptibility and (ii) antagonistic activity against poultry-associated pathogens (*Salmonella* Typhimurium, *Salmonella* Enteritidis, *Escherichia fergusonii*, and *Listeria monocytogenes*). Susceptibility was first assessed using complementary agar disk diffusion screening with 14 antibiotics at different concentrations and minimum inhibitory concentration (MIC) determination for seven antibiotics using Etest®. Subsequently, cell-free supernatants were evaluated using microtiter plate pathogen growth inhibition assays. The results revealed pronounced strain- and antibiotic-specific variability in observed antibiotic susceptibility profiles, underscoring the importance of strain-level assessment and cautious interpretation in the absence of standardized breakpoints for many probiotic species. All untreated cell-free supernatants produced strong, statistically significant in vitro growth inhibition of the four pathogens: Gram-negative *S.* Typhimurium, *S.* Enteritidis, and *E. fergusonii*, and Gram-positive L. *monocytogenes*.

Enzyme-treatment experiments suggested that peroxide-sensitive and, in some strain–pathogen combinations, lipase-sensitive components contributed to antagonism; however, the pattern was strain- and pathogen-dependent and is therefore consistent with a multifactorial inhibitory effect rather than a single dominant mechanism.

Overall, this study highlights the value of integrating antibiotic susceptibility profiling with functional antagonism assays for rational prioritization of candidate probiotic strains. The results identify candidate probiotics for further genomic, mechanistic, and in vivo validation rather than supporting stand-alone safety or efficacy claims.

## 1 Introduction

The global poultry industry faces the dual challenge of sustaining high productivity while reducing reliance on antibiotics in the context of escalating antimicrobial resistance (AMR). More than 100,000 tons of antimicrobials are estimated to be used annually in animal husbandry, with poultry production accounting for a substantial proportion of this use [1], [2]. Poultry systems are also recognized as important reservoirs of multidrug resistance (MDR), including resistance to critically important antimicrobials for human medicine, such as fluoroquinolones, β-lactams, polymyxins, macrolides, and carbapenems [3], [4] Continued antimicrobial exposure in animal production contributes to the persistence and dissemination of multidrug-resistant bacteria across animal, environmental, and food-chain interfaces, which has intensified interest in One Health strategies capable of reducing antibiotic dependence, while maintaining animal health and food safety.

One promising approach is the use of probiotics as functional feed additives. Probiotics are defined as ‘live microorganisms that, when administered in adequate amounts, confer a health benefit on the host’ [5]. In poultry, commonly studied probiotic taxa include *Lactobacillus* spp., *Bacillus* spp., *Enterococcus faecium*, *Bifidobacterium* spp., and *Pediococcus* spp., selected for traits such as gastrointestinal survival, interaction with the intestinal epithelium, and production of antimicrobial or immunomodulatory metabolites [6], [7], [8]. Apart from using probiotics directly as supplements, new research has brought to light the benefits of using fermentation metabolites and bioactive ingredients as feed additives that can be used to manage the intestinal microbiota in poultry. These can include feed fermentation, the use of postbiotics, or combinations of probiotics and prebiotics in an effort to provide a synergistic effect on intestinal barrier function, immune response, and colonization resistance against enteric pathogens [9], [10].

In addition to immunological effects, probiotics may improve nutrient utilization and growth performance in poultry, and these benefits are generally attributed to competitive exclusion of pathogens, modulation of host responses, and secretion of inhibitory metabolites such as organic acids, hydrogen peroxide, bacteriocins, and related bioactive compounds [11], [12], [13], [14]. Accordingly, probiotics are increasingly being considered as part of antibiotic-reduction strategies in commercial poultry production and broader food chain health management.

Early-life probiotic administration is of particular interest because it may help to shape the developing gut microbiota before stable pathogen colonization is established. In broilers, early supplementation, including *in ovo* delivery, has been associated with improved mucosal immunity, stabilization of gut microbiota, and reduced colonization by enteric pathogens such as *Salmonella enterica*, *Escherichia coli*, and *Clostridium perfringens* [15], [16], [17], [18]. More broadly, early-life microbiota modulation has also been linked to lower AMR gene burden in developing gut ecosystems, supporting the rationale for preventive microbiota-based interventions at the beginning of life [19], [20], [21]. However, probiotic efficacy is strain-dependent and context-specific, which makes careful selection and characterization of candidate strains essential before application. The strains investigated in the present study were selected to represent several probiotic groups relevant to poultry systems, namely *Lactobacillus* spp., *Bifidobacterium lactis*, *Carnobacterium* spp., and *Bacillus* spp. The latter have technological advantages for animal production because their spores can survive feed processing and passage through the gastrointestinal tract [22], [23], [24], [25], [26], [27]. In our previous work, the *Lactobacillus* and *Bacillus* strains included here were also compatible with selected candidate prebiotics intended for *in ovo* synbiotic application, supporting their further evaluation as poultry-relevant candidates [23], [28] This combination of biological relevance and practical applicability make these strains suitable for an initial comparative screen, particularly in the context of early-life synbiotic strategies.

Selection of appropriate poultry-associated and foodborne indicator pathogens is equally important. *Salmonella* Typhimurium and *Salmonella* Enteritidis remain leading zoonotic agents associated with poultry meat and eggs and are frequently carried asymptomatically by birds, facilitating persistence and transmission within production systems [29], [30], [31], [32]. *Escherichia fergusonii* has emerged as an opportunistic avian pathogen linked to colibacillosis in poultry and has been reported to harbor transferable resistance determinants, including *mcr-1* [33], [34]. Moreover, *E. fergusonii* can confound traditional *E. coli* diagnostic assays in poultry operations because of phenotypic and genotypic similarities, underscoring its epidemiological importance in flock health monitoring and antimicrobial-stewardship programs. Although *Listeria monocytogenes* is less commonly associated with disease in live poultry, it is relevant to poultry-processing environments and carcass contamination and therefore remains important from a food safety perspective. These bacteria were therefore selected as poultry-associated and foodborne indicator pathogens for evaluation of probiotic antagonistic potential. Together, they span both Gram-negative and Gram-positive bacteria relevant to poultry production and food safety. For probiotics intended for use as feed additives, antimicrobial susceptibility testing serves primarily as a safety assessment. Candidate strains should be characterized not only for antagonistic activity but also for their susceptibility profiles, particularly with respect to veterinary-relevant antimicrobial classes such as tetracyclines, macrolides, aminoglycosides, and polymyxins [35], [36], [37]. Their safety evaluation also has a regulatory dimension. Within the European Food Safety Authority (EFSA) framework for microorganisms used in the food/feed chain, assessment is conducted at strain level and relies on phenotypic minimum inhibitory concentration (MIC)-based susceptibility testing together with genome-based interrogation for known AMR determinants [38], [39], [40]. Within the Qualified Presumption of Safety (QPS) approach, bacterial strains should not harbor acquired genes conferring resistance to therapeutic antimicrobials, and *Bacillus* spp. must also lack toxigenic potential. Accordingly, the present work should be considered an initial in vitro screening step relevant to candidate prioritization rather than a complete regulatory safety assessment. Against this background, the present study performed a targeted in vitro screening of selected candidate probiotic strains, focusing on two key criteria: (i) antagonistic activity against poultry-associated and foodborne indicator pathogens and (ii) observed antimicrobial susceptibility profiles relevant to safety assessment. We hypothesized that the tested strains would display strain-dependent antagonistic effects against *S.* Typhimurium, *S.* Eenteritidis, *E. fergusonii*, and *L. monocytogenes*, together with heterogeneous antibiotic susceptibility patterns that would support prioritization of candidates for further study. Accordingly, the objectives were: (i) to assess antibiotic susceptibility profiles of 10 candidate probiotic strains using complementary diffusion-based screening and MIC-based approaches; (ii) to evaluate the in vitro antagonistic activity of these strains against four poultry-associated and foodborne indicator pathogens; and (iii) to identify promising probiotic candidates for further mechanistic, molecular, and in vivo validation as microbiota-based tools for poultry production within a One Health framework.

## 2 Materials and methods

The experimental design used in the current study is presented in Fig. 1.

**Fig. 1:**
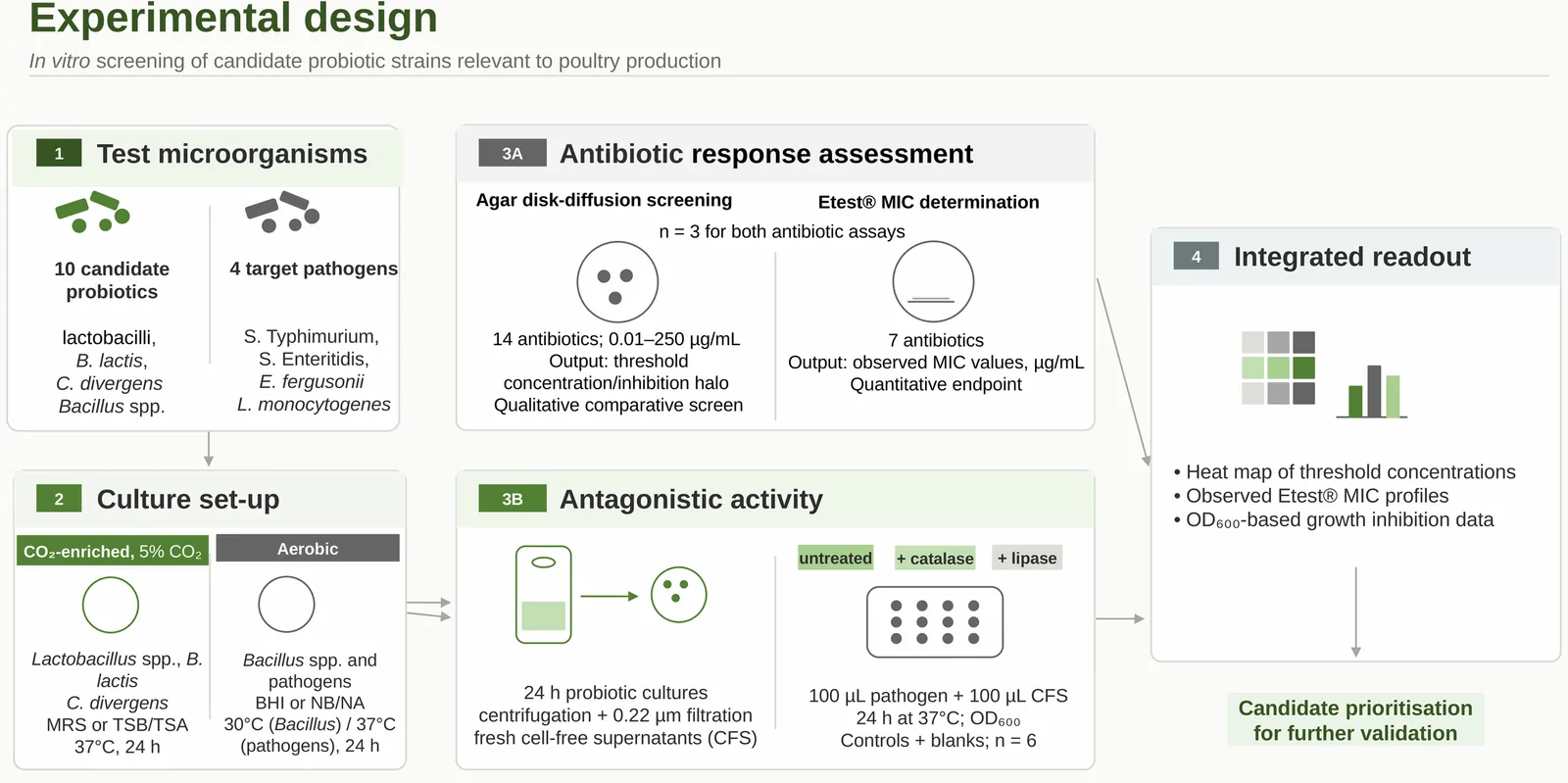
Experimental design of the in vitro screening workflow for candidate probiotic prioritization. Ten candidate probiotic strains and four poultry-associated and foodborne pathogens were included. Candidate strains were cultured under CO₂-enriched or aerobic conditions, as appropriate, and evaluated using two complementary antibiotic-response workflows: qualitative agar disk-diffusion screening with 14 antibiotics and quantitative Etest® MIC determination with seven antibiotics. In parallel, fresh cell-free supernatants were tested untreated or after catalase/lipase treatment for OD₆₀₀-based pathogen growth inhibition. The integrated readout was used to support candidate prioritization for further validation.

### 2.1 Microorganisms used in the study and culture conditions

Ten candidate probiotic strains were evaluated for antibiotic susceptibility and antagonistic activity: *Lactobacillus plantarum* ATCC 11974 (L. plant 1), *Bifidobacterium lactis* NCC2818 (B. lact), *L. rhamnosus* H25 (L. rham), *L. plantarum* strain 2 (L. plant 2) and *Carnobacterium divergens* (C. diverg), *Bacillus altitudinis 1* (B. altit 1), *B. altitudinis* 2 (B. altit 2), *B. pumilus* 1 (B. pumil 1), *B. pumilus* 2 (B. pumil 2) and *B. pumilus* 3 (B. pumil 3). Strains were selected based on published evidence of beneficial properties relevant to poultry and/or humans, including suitability for the target host (poultry), feasibility of *in ovo* application, prior use in synbiotic formulations, beneficial modulation of the host microbiome, and reported antagonistic potential. Details of the selection concept and strain acquisition/origin were described previously [23], [28] and are summarized in Table 1, Table 2.

**Table 1:** Candidate probiotic test strains used in the study.

Strain code used in manuscript	Species	Strain designation	Source	Culture conditions	Key characteristics relevant to study specific to the strain/species	Justification for selection
L. plant 1	*Lactobacillus plantarum*	ATCC 11974	ATCC	MRS, 37 °C, 5% CO₂	Stress tolerance (acid, bile, temperature, osmotic) and strong adhesion to intestinal cells [41]	Conserved probiotic traits and immunostimulatory capacity suitable for *in ovo* delivery [[42], [43], [44]]
B. lact	*Bifidobacterium lactis*	NCC2818	NCC	MRS, 37 °C, 5% CO₂	Stimulation of cytokine/immunoglobulin production and intestinal barrier function in newborn models [[45], [46]]	Well-characterized probiotic for early life mucosal immunity evaluation [47]
L. rham	*Lactobacillus rhamnosus*	H25	DISTAAM collection of the University of Molise [48]	MRS, 37 °C, 5% CO₂	Strong epithelial adhesion and stimulation of IgA-producing cells and cytokine responses in poultry [43]	*Lactobacillus* was used earlier for in *in ovo*/early post-hatch treatment with intact viability of the embryos [[48], [49], [50], [51], [52]]
L. plant 2	*Lactobacillus plantarum*	MT613640 Accession number: OP804340 [28]	DISTAAM collection of the University of Molise	MRS, 37 °C, 5% CO₂	Tolerance to bile/acids; production of antimicrobial compounds for early gut colonization [43]	Proven strain-specific *L. plantarum* colonization and immunomodulatory effects after *in ovo* delivery [[53], [54]]
C. diverg	*Carnobacterium divergens*	MN229536 Accession number: OP804339 [28]	Isolated and described by the JHJ company in Poland	TSB/TSA, 37 °C, 5% CO₂	Colonization of chicken gut and shaping early-life microbial communities [[14], [47]]	Slow-growing LAB suitable for underdeveloped GIT and *in ovo* synbiotic formulations [[28], [55]]
B. altit 1	*Bacillus altitudinis*	N/A	Seaweed (*Fucus vesiculosus)*	BHI, 30 °C, aerobic	Tolerance to bile; antimicrobial activity; adherence to intestinal epithelial cells [[25], [56]]	Spore formation and in vitro probiotic properties [57]
B. altit 2	*Bacillus altitudinis*	N/A	Seaweed (*Fucus serratus)*	BHI, 30 °C, aerobic	Antimicrobial activity; adherence to intestinal epithelial cells [[25], [56]]	Spore formation and in vitro probiotic properties; An *B. altitudinis* strain fed to chickens reduced *Eimeria* infection, increased intestinal expression of tight junction proteins and cytokines and modulated the intestinal microbiota; [57]
B. pumil 1	*Bacillus pumilus*	N/A	Seaweed (*Ulva* spp.*)*	BHI, 30 °C, aerobic	Tolerance to simulated gastric transit; antimicrobial activity; adherence to intestinal epithelial cells [[25], [56]]	Spore formation and in vitro probiotic properties; *B. pumilus* strains fed to chickens increased body weight and feed intake and increased expression of tight junction proteins in the ileum [[47], [58]] and alleviated inflammatory injury caused by LPS and attenuated the inflammatory response [59]
B. pumil 2	*Bacillus pumilus*	N/A	Seaweed (*Palmaria palmata*)	BHI, 30 °C, aerobic	Antimicrobial activity; adherence to intestinal epithelial cells [[25], [56]]	Spore formation and in vitro probiotic properties; *B. pumilus* strains fed to chickens increased body weight and feed intake and increased expression of tight junction proteins in the ileum [47] and alleviated inflammatory injury caused by LPS and attenuated the inflammatory response [59]
B. pumil 3	*Bacillus pumilus*	N/A	Seaweed (*Ulva* spp.*)*	BHI, 30 °C, aerobic	Tolerance to simulated gastric transit and bile; antimicrobial activity; adherence to intestinal epithelial cells [[25], [56]]	Spore formation and in vitro probiotic properties; *B. pumilus* strains fed to chickens increased body weight and feed intake and increased expression of tight junction proteins in the ileum [58] and alleviated inflammatory injury caused by LPS and attenuated the inflammatory response [60]

Abbreviations: ATCC - American Type Culture Collection; NCC – Nestlé Culture Collection; DISTAAM- Dipartimento di Scienze e Tecnologie Agro-alimentari, Ambientali e Microbiologiche (Department of Agri-food, Environmental and Microbiological Sciences) at the University of Molise, Italy; TSB - Tryptic Soy Broth; TSA - Tryptic Soy Agar; BHI - Brain Heart Infusion; LAB – Lactic Acid Bacteria; GIT – Gastrointestinal Tract; MRS - De Man, Rogosa, and Sharpe; LPS – lipopolysaccharide; N/A – not applicable.

**Table 2:** Poultry-associated and foodborne pathogen strains used in the study.

Species/serotype	Strain designation	Source	Culture conditions	Relevance to poultry/food safety specific for the species	Justification for selection
*Salmonella* Typhimurium	ATCC 14028	ATCC	NB/NA, 37 °C, aerobic	Causes systemic infection; standard poultry challenge strain [61]. Standard to test probiotic protection against early host-pathogen interactions in *in ovo* systems [29]	Used to assess probiotic protection against early host-pathogen interactions [29]
*Salmonella* Enteritidis	ATCC 13076	ATCC	NB/NA, 37 °C, aerobic	Primary cause of egg-borne *salmonellosis* and vertical transmission [[62], [63]]	Model for challenges in early gut colonization relevant to egg safety and probiotic antagonism [[64], [65]]
*Escherichia fergusonii*	ATCC 35469	ATCC	NB/NA, 37 °C, aerobic	APEC causes colibacillosis in poultry with multidrug resistance [64]	*E. fergusonii* isolates recovered from pigs, chickens, and ducks, had sulfonamide and tetracycline resistance phenotypes; this species is directly relevant in the context of antibiotic reduction strategies [29]
*Listeria monocytogenes*	ATCC 7644	ATCC	BHI broth/agar, 37 °C, aerobic	Crosses gut barrier; relevant to poultry processing contamination [[65], [66]]	Gram-positive invasive pathogen [[67], [68]]

Abbreviations: ATCC – American Type Culture Collection; NA- Nutrient Agar; NB – Nutrient Broth; BHI - Brain heart infusion; APEC - Avian Pathogenic *Escherichia coli*.

*Lactobacillus* spp. and *B. lactis* were routinely cultured in de Man, Rogosa and Sharpe (MRS) broth/agar (Millipore, Poznań, Poland), and *C. divergens* was cultured in Tryptic Soy Broth/Tryptic Soy Agar (TSB/TSA) (Millipore, Poznań, Poland) at 37 °C for 24 h. Incubation was performed under reduced‑oxygen conditions using a CO₂ incubator (5% CO₂). Since 5% CO₂ does not constitute strict anaerobiosis, these incubation conditions are referred to hereafter as CO₂-enriched conditions. *Bacillus* strains were cultivated aerobically in Brain Heart Infusion (BHI) broth and on BHI agar (Merck Millipore/Sigma-Aldrich, Poznań, Poland) at 30 °C for 24 h. Four poultry-associated and foodborne pathogens obtained from the American Type Culture Collection (ATCC) were used: *S.* Typhimurium ATCC 14028, *S.* Enteritidis ATCC 13076, *E. fergusonii* ATCC 35469, and *L. monocytogenes* ATCC 7644. Pathogens were cultured in nutrient broth/agar (Merck Millipore), except for L. *monocytogenes*, which was grown in BHI broth/agar, at 37 °C for 24 h.

### 2.2 Antibiotic susceptibility assessment of probiotic strains

Antibiotic response was assessed using two complementary but distinct approaches. First, a qualitative agar disk-diffusion screening assay was performed with 14 powdered antibiotics at seven tested concentrations to compare inhibition patterns among candidate probiotic strains. This assay was used to record visible inhibition halos and threshold concentrations, defined as the lowest antibiotic concentration loaded onto the disc that produced a visible inhibition halo under the test conditions. It was not used to determine MIC values nor to assign categorical susceptibility or resistance. Secondly, quantitative MIC determination was performed using commercial Etest® gradient strips for seven selected antibiotics. Etest® results are reported as observed MIC endpoints expressed in μg/mL. Antibiotics were selected to cover major antimicrobial classes relevant to veterinary and human medicine and to permit cross-strain comparison in an exploratory screen (Table 3). The panel was not intended as a formal EFSA-compliant susceptibility-assessment panel, and EFSA-recommended quality-control strains were not included.

**Table 3:** Antibiotics used in the study and their characteristics. The table shows the full antimicrobial panel, including 14 antibiotics used for disk-diffusion screening and seven antibiotics used for Etest® MIC determination.

Antibiotic name	Abbreviation	Diffusion method	*E*-test	Class	Action	Effect	References
Amoxicillin	AM	X	X	Beta-lactam penicillins	Bactericidal	Inhibition of bacterial cell wall synthesis	[[69], [70]]
Ampicillin	Amp		X	Beta-lactam penicillins	Bactericidal	Inhibition of bacterial cell wall synthesis	[[69], [71]]
Clavulanic acid	C	X		Beta-lactamase inhibitor	Bactericidal in combination	Inactivation of *β*-lactamase enzymes	[[72], [73]]
Chlortetracycline	ChT	X		Tetracyclines	Bacteriostatic	Inhibition of protein synthesis by binding to the 30S ribosomal subunit	[[74], [75]]
Ciprofloxacin	Cl	X	X	Fluoroquinolones	Bactericidal	Inhibition of DNA synthesis	[[69], [76]]
Erythromycin	EM	X	X	Macrolides	Bacteriostatic	Inhibition of protein synthesis	[77]
Furazolidone	Fu	X		Nitrofuran	Bactericidal/bacteriostatic depending on concentration	Formation of reactive nitro-reduction products that damage bacterial macromolecules and interfere with bacterial enzyme systems	[78]
Gentamicin	GM	X	X	Aminoglycoside	Bactericidal	Inhibition of protein synthesis by binding to the 30S ribosomal subunit and causing misreading	[79]
Penicillin	P	X		Penicillins	Bactericidal	Cell wall disruption by preventing peptidoglycan cross-linking	[[80], [81]]
Rifampicin	RI	X	X	Ansamycin	Bactericidal	Blockage of bacterial transcription	[[82], [83]]
Streptomycin	S	X		Aminoglycoside	Bactericidal	Inhibition of protein synthesis by binding to the 30S ribosomal subunit and causing misreading	[[84], [85]]
Spiramycin	Sp	X		Macrolide	Bacteriostatic	Inhibition of protein synthesis by binding to the 50S ribosomal subunit	[86]
Tylosin	Ty	X		Macrolide	Bacteriostatic	Inhibition of protein synthesis by binding to the 50S ribosomal subunit	[87]
Tetracycline	Tet		X	Tetracycline	Bacteriostatic	Inhibition of protein synthesis by binding to the 30S ribosomal subunit	[75]
Vancomycin	Va	X		Glycopeptide	Bactericidal	Inhibition of cell wall synthesis by binding to precursors	[69]
Virginiamycin	Vi	X		Streptogramin	Bactericidal (synergistic)	Two antibiotic components synergistically inhibit protein synthesis at the 50S subunit	[88]

Where relevant, the observed results were interpreted in the context of available reference standards and guidance documents, including European Committee on Antimicrobial Susceptibility Testing (EUCAST) breakpoint tables and disk diffusion methodology, Clinical and Laboratory Standards (CLSI) reference standards for disk diffusion and dilution antimicrobial susceptibility testing, and EFSA guidance on microorganism characterization in the food/feed chain [[40], [89], [90], [91], [92], [93]]. However, because the present assays were not performed under fully standardized EUCAST/CLSI conditions and validated breakpoints are unavailable for many probiotic species and test formats; results are reported here as observed threshold concentrations and observed Etest® MICs rather than as categorical susceptibility assignments. Because 10 μL was applied to each 5-mm disc in the disk-diffusion screening, the experimental variable was the antibiotic mass per disc. However, biological exposure was not directly comparable among antibiotics because diffusion characteristics differ between compounds.

#### 2.2.1 Qualitative agar disk-diffusion screening with 14 powdered antibiotics

Fourteen antibiotics were used for disk-diffusion screening: chlortetracycline (ChT), rifampicin (RI), vancomycin (V), streptomycin (S), virginiamycin (Vi), ciprofloxacin (CI), clavulanic acid (C), penicillin (P), gentamicin (GM), amoxicillin (AM), spiramycin (SP), tylosin (TY), furazolidone (Fu), and erythromycin (EM) (all obtained from Sigma-Aldrich). Fresh probiotic cultures were adjusted to McFarland 1.0 and spread onto agar plates appropriate for each organism (MRS for *Lactobacillus* and *B. lactis*, TSA for *C. divergens*, and BHI for *Bacillus*). Sterile self-prepared blank paper discs (5-mm diameter) were loaded with 10 μl of antibiotic working solutions at the following concentrations: 0.01, 25, 50, 100, 150, 200, and 250 μg/mL. Powdered antibiotics were dissolved in the appropriate solvents to prepare stock solutions as follows: sterile distilled water for water-soluble agents (vancomycin, streptomycin, ciprofloxacin, clavulanic acid, penicillin, gentamicin, amoxicillin); 50–70% ethanol for chlortetracycline, virginiamycin, spiramycin, tylosin, and erythromycin; and methanol or DMSO (dimethyl sulfoxide) for rifampicin and furazolidone. Working solutions were prepared by diluting stock solutions in sterile distilled water to the tested concentrations. Clavulanic acid was included because of its role as a β-lactamase inhibitor and its relevance to β-lactam resistance contexts; however, because it was tested alone, its results were interpreted only as exploratory inhibition observations. Negative controls were prepared in parallel by loading sterile 5-mm paper discs with 10 μL of the corresponding solvent/vehicle used for each antibiotic, prepared in the same way as the antibiotic working solutions but without antibiotic. Both antibiotic-loaded and vehicle-control discs were allowed to absorb and air-dry for 10–15 min in a laminar-flow cabinet before placement on inoculated agar plates to minimize residual solvent effects. No inhibition halos were observed around the control discs, confirming that the solvents or disc material did not contribute to growth inhibition under the experimental conditions. Antibiotic concentrations (0.01–250 μg/mL) were selected to span a broad exposure range across the tested antibiotics, informed by published broth microdilution studies and related antimicrobial susceptibility testing literature [[90], [94], [95], [96]]. *Lactobacillus* spp., *B. lactis* and *C. divergens* were incubated under CO₂-enriched conditions at 37 °C for 24 h, whereas *Bacillus* strains were incubated aerobically at 30 °C for 24 h. For each strain-antibiotic concentration, the threshold concentration was defined as the lowest antibiotic concentration loaded onto the disc that produced a visible inhibition halo under the test conditions. Each combination was tested in triplicate.

#### 2.2.2 Quantitative Etest® MIC determination with seven antibiotics

Etest® gradient strips (BioMérieux, Warsaw, Poland) were used for quantitative MIC determination on agar media. The MICs were determined for seven antibiotics: gentamicin (GM), ciprofloxacin (Cl), tetracycline (Tet), amoxicillin (AM), ampicillin (Amp), erythromycin (EM), and rifampicin (RI). The procedure used was performed according to the manufacturer's instructions, using the same media and incubation conditions as described above for each candidate probiotic strain. The MICs were read at the point where the inhibition ellipse intersected the Etest® strip and are reported as observed MIC endpoints in μg/mL. Each probiotic–antibiotic combination was tested in triplicate.

### 2.3 Antagonistic activity of probiotics

Test strains were individually cultured in 25 mL of appropriate broth (MRS for *Lactobacillus* spp. and *B. lactis*; BHI for *Bacillus* spp. and *C. divergens*) in 50 mL flasks. Cultures were inoculated from single colonies and incubated statically at strain-specific temperatures (30 °C or 37 °C) for 24 h. On day 2, cultures were centrifuged at 10,000 rpm for 5 min at 4 °C, and supernatants were collected and filtered through 0.22 μm Titan3 PES syringe filters (Alchem, Wrocław, Poland) to obtain cell-free supernatants (CFS). Each biological replicate was prepared from an independently inoculated culture.

The CFS from the test strains were assessed against the pathogens *S.* Typhimurium, *S.*Enteritidis, *E. fergusonii*, and *L. monocytogenes*, using a microtiter plate growth-inhibition assay as follows. Poultry-associated and foodborne pathogen cultures were adjusted to McFarland 0.5 (∼1 × 10^8^ CFU/mL) and diluted 1:2 in the assay, resulting in a final inoculum of ∼5 × 10^7^ CFU/mL per well. Inoculum densities were standardized using McFarland standards, which is common practice in similar CFS-based antagonism assays, and viable counts were not performed for each replicate. This inoculum exceeds the standard CLSI/EUCAST broth microdilution recommendation of 5 × 10^5^ CFU/mL and was intentionally selected for two reasons: (i) to model a high-challenge scenario that may occur under intensive poultry production conditions, thereby providing a stringent test of probiotic antagonistic capacity; and (ii) to maintain methodological consistency with Choi et al. [97], who used a similar inoculum density (McFarland 0.5) in microtiter plate growth-inhibition assays with CFS.

In each well, 100 μL pathogen culture (24 h cultures in nutrient broth or BHI for *L. monocytogenes*, adjusted to McFarland 0.5 i.e. ∼10^8^ CFU/mL) was combined with 100 μL of one of the following: (i) untreated CFS, (ii) CFS treated with catalase (1 mg/mL; Sigma-Aldrich; 37 °C, 1 h), (iii) CFS treated with lipase (1 mg/mL; Sigma-Aldrich; 37 °C, 1 h), as per Choi et al. [97]. Control wells contained pathogen inoculum plus sterile nutrient broth/BHI broth to match the corresponding CFS (growth control). Uninoculated nutrient broth/BHI broth blanks were included for background subtraction. All treatments were tested in six replicates. After 24 h incubation at 37 °C, pathogen growth was determined by measuring OD_600_ using a SpectraMax® ID3 Multi-Mode Microplate Reader (Molecular Devices®, Warsaw, Poland). OD_600_ was used to quantify pathogen growth because the assay was designed as a high-throughput growth-inhibition screen, where optical density provides a reliable, linear proxy for pathogen growth under these conditions, consistent with previous studies.

The CFS were additionally treated with catalase (1 mg/mL; Sigma-Aldrich; 37 °C, 1 h) or lipase (1 mg/mL; Sigma-Aldrich; 37 °C, 1 h), as per Choi et al. [97]. Enzyme activities were not directly assayed after treatment; instead, we relied on established protocols and supplier recommendations and included appropriate controls (untreated CFS and enzyme-treated CFS) to interpret the effects.

The pH of the untreated CFS was measured before use in the antagonism assay. The final pH values of the untreated CFS ranged from ∼3.8 to 4.5 for the lactic acid bacteria and *C. divergens*, and from ∼6.0 to 7.0 for the *Bacillus* strains.

### 2.4 Statistical analyses

All results are presented as mean ±SD. The number of replicates per experiment is specified above: disk diffusion screening (*n* = 3 per antibiotic concentration); Etest® (*n* = 3); antagonism assay (*n* = 6 per treatment). For disk diffusion screening and Etest® assays, each replicate was prepared from an independently inoculated culture (biological replicates) and threshold concentrations were assigned and visualized as heat maps prepared in R (version 4.3.1) using ggplot2 package. Etest® MIC values were tested by Levene's and Shapiro-Wilk's test. Normality of distribution and homogeneity of variance were tested separately for each antibiotic–strain combination using the Shapiro–Wilk and Levene's tests, respectively. Because the assumptions of normal distribution were not met for Etest® MIC values, differences between bacteria for each antibiotic were tested with the Kruskal–Wallis test followed by Dunn's post hoc test with adjustment for multiple comparisons. For the antagonism assay, comparisons of poultry-associated and foodborne pathogen growth across treatments (control vs CFS variants) were conducted using one-way ANOVA followed by Tukey's HSD post hoc test (*p* < 0.05) separately for each pathogen–probiotic combination. Statistical analyses were performed using Statistica 13.3 (StatSoft, Kraków, Poland).

## 3 Results

### 3.1 Antibiotic susceptibility screening using the agar disk diffusion assay to determine threshold concentrations

The results of antibiotic susceptibility screening of the 10 test strains against 14 antibiotics at different concentrations using agar disk diffusion assays are summarized in Fig. 2. This screening approach provides a comparative overview of growth inhibition patterns among the strains of *Lactobacillus*, *Carnobacterium*, and *Bacillus* based on diffusion-based assays, rather than MIC determination. Distinct strain- and antibiotic-dependent differences in inhibition patterns were observed. *Lactobacillus* isolates generally were only weakly inhibited or not inhibited in the presence of several antibiotics, including vancomycin (Va), clavulanic acid (C), gentamicin (GM), streptomycin (S), ciprofloxacin (Cl) and furazolidone (Fu), as indicated by the high threshold concentrations, whereas pronounced growth inhibition was observed for virginiamycin (Vi) and spiramycin (Sp), as indicated by the low threshold concentrations recorded for several strains. *L. plantarum* strain 1, but not L. *plantarum* strain 2, was relatively broadly inhibited by high concentrations of multiple antibiotics in this screening assay, indicating lower sensitivity than the other test strains under disk diffusion-based conditions. The *C. divergens* displayed inhibition patterns comparable to those of *Lactobacillus* spp., particularly in response to virginiamycin and spiramycin. In contrast, the *Bacillus* spp. exhibited more varied inhibition patterns across the tested antibiotics, although all isolates showed visible inhibition at low threshold concentrations of amoxicillin (AM), ciprofloxacin (Cl), erythromycin (EM), gentamicin (GM), penicillin (P), rifampicin (RI), tylosin (Ty) and vancomycin (Va), indicating that they were sensitive to these antibiotics. In contrast, the threshold concentrations for clavulanic acid (C), furazolidone (Fu) and streptomycin (S) were much higher under the tested diffusion conditions. Notably, *B. pumilus* 3 and *B. altitudinis* 2 showed low thresholds for virginiamycin in this screening assay. Overall, this diffusion-based screening highlights pronounced strain-dependent variability in antibiotic response patterns and supports the use of complementary MIC determination methods (e.g., Etest®) for a more detailed characterization of antibiotic susceptibility in probiotic candidates.

**Fig. 2:**
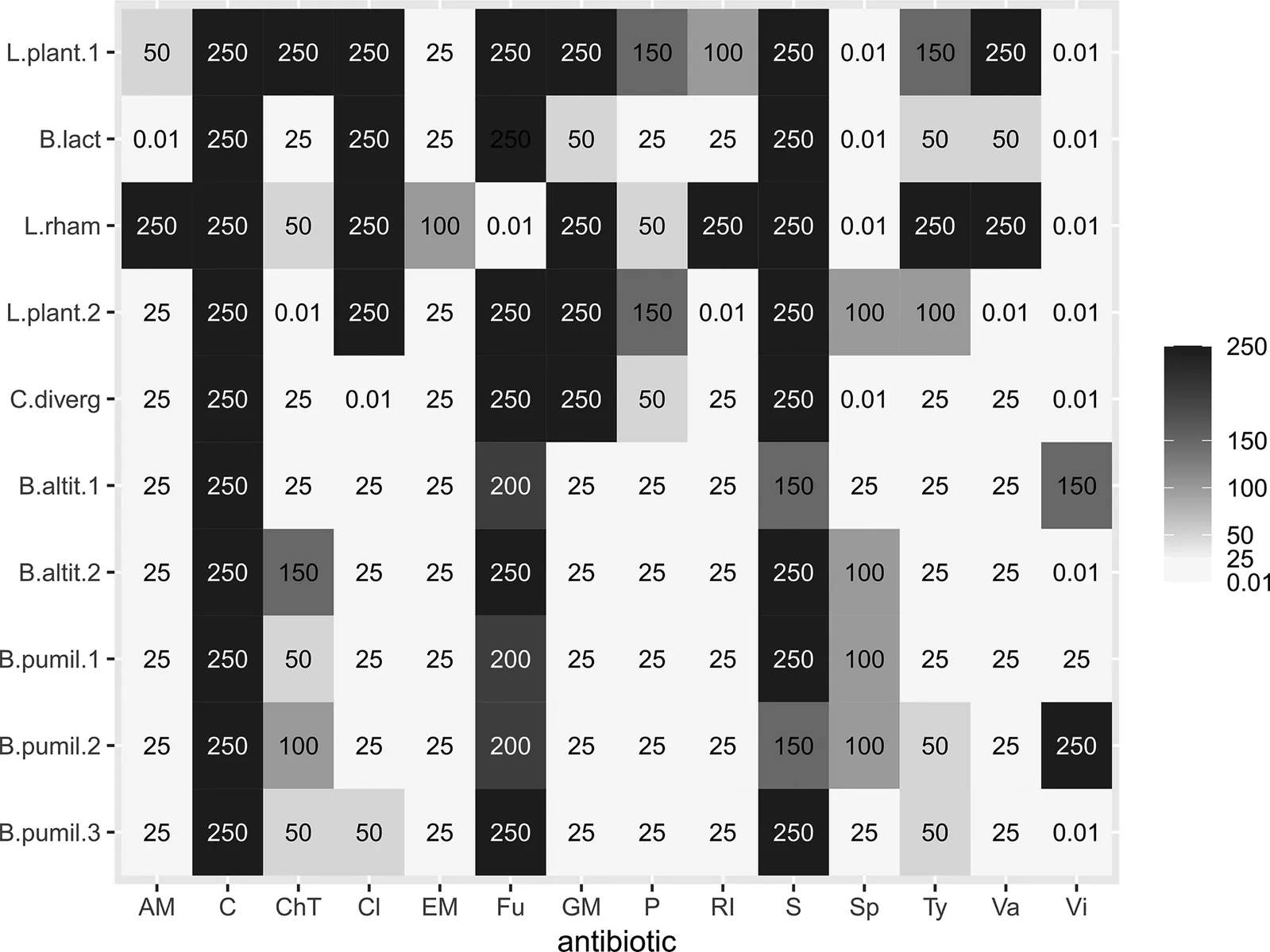
Heat map of antibiotic susceptibility screening by agar diffusion method. The threshold concentration was defined as the lowest antibiotic concentration loaded onto the disc that produced a visible inhibition halo under the test conditions (0.01–250 μg/mL) (*n* = 3)^1^. ^1^Higher threshold concentrations indicate that a higher amount of antibiotic was required before visible inhibition was observed, consistent with lower susceptibility under the assay conditions. The shading represents categories of growth inhibition observed in agar diffusion screening. This assay was used for comparative screening and was not used for MIC determination or categorical susceptibility assignment. Abbreviations: L. plant 1 - *Lactobacillus plantarum* ATCC 11974, B. lact - *Bifidobacterium lactis* NCC2818, L. rham - *Lactobacillus rhamnosus* H25, L. plant 2 - *Lactobacillus plantarum*, C. diverg - *Carnobacterium divergens*, B. altit 1 - *Bacillus altitudinis* 1, B. altit 2 - *B. altitudinis* 2, B. pumil 1 - *Bacillus pumilus* 1, B. pumil 2 - *B. pumilus* 2, B. pumil 3 - *B. pumilus* 3; AM – amoxicillin, C – clavulanic acid, ChT – chlortetracycline, Cl – ciprofloxacin, EM – erythromycin, Fu – furazolidone, GM – gentamicin, P – penicillin, RI -rifampicin, S – streptomycin, Sp – spiramycin, Ty – tylosin, Va – vancomycin, Vi – virginiamycin.

### 3.2 Antibiotic susceptibility of test strains assessed by Etest® (observed MIC values)

Observed MIC values determined by Etest® varied substantially across test strains and antibiotics (Table 4), indicating pronounced strain- and antibiotic-dependent differences in susceptibility profiles. In this study, MIC values are reported as observed Etest® MICs; categorical interpretation (e.g., “susceptible/resistant”) should only be applied where validated breakpoints are available for the relevant species/antibiotic/method. Because validated breakpoints are not available for the Etest® format for most taxa assessed here, the values are reported as observed Etest® endpoints and should not be interpreted as EFSA-compliant susceptibility classifications [38].

**Table 4:** Minimum inhibitory concentrations (MICs, μg/mL) of seven antibiotics for 10 strains of probiotic bacteria determined using Etest® (means of 3 assays, SD in brackets).

	Antibiotic						
Probiotic	GM	Cl	Tet	AM	Amp	EM	RI
L. plant 1	2.67 (0.58) ^ab^	*	*	0.02 (0.00) ^b^	0.08 (0.02) ^ab^	1.00 (0.00) ^ab^	1.70 (0.04) ^ab^
B. lact	0.00	*	4.00 (0.00) ^ab^	0.50 (0.00) ^ab^	1.16 (0.72) ^ab^	6.00 (0.00) ^a^	0.83 (0.14) ^ab^
L. rham	7.33 (1.15) ^ab^	*	*	0.00	2.00 (0.00) ^b^	4.00 (0.00) ^ab^	0.29 (0.18) ^ab^
L. plant 2	8.00 (0.00) ^ab^	*	*	0.16 (0.00) ^ab^	0.19 (0.00) ^ab^	3.33 (1.15) ^ab^	0.19 (0.00) ^ab^
C. diverg	12.00 (0.00) ^b^	*	1.50 (0.00) ^b^	0.04 (0.02) ^b^	0.02 (0.00) ^a^	0.05 (0.00) ^b^	12.00 (0.00) ^b^
B. altit 1	1.33 (0.29) ^ab^	24.00 (0.00) ^ab^	12.00 (0.00) ^a^	3.00 (0.00) ^a^	1.33 (0.58) ^ab^	0.83 (0.29) ^ab^	0.54 (0.19) ^ab^
B. altit 2	2.00 (0.00) ^ab^	32.00 (0.00) ^a^	8.00 (0.00) ^ab^	1.83 (0.29) ^ab^	0.46 (0.07) ^ab^	2.00 (0.00) ^ab^	*
B. pumil 1	1.50 (0.00) ^ab^	1.50 (0.00) ^b^	*	2.00 (0.00) ^ab^	0.58 (0.14) ^ab^	3.00 (0.00) ^ab^	0.67 (0.29) ^ab^
B. pumil 2	1.00 (0.00) ^a^	*	*	0.83 (0.14) ^ab^	0.13 (0.00) ^ab^	2.33 (0.58) ^ab^	0.21 (0.03) ^ab^
B. pumil 3	1.50 (0.00) ^ab^	3.00 (0.00) ^ab^	*	1.33 (0.29) ^ab^	1.00 (0.50) ^ab^	24.00 (0.00) ^a^	2.00 (0.00) ^a^

^a,b^ Within each column, values that do not share a common superscript are significantly different (Kruskal–Wallis test followed by Dunn's post hoc test, *p* ≤ 0.05). * - < lowest concentration on the Etest® strip.

Abbreviations: L. plant 1 - *Lactobacillus plantarum* ATCC 11974, B. lact - *Bifidobacterium lactis* NCC2818, L. rham - *Lactobacillus rhamnosus* H25, L. plant 2 - *Lactobacillus plantarum*, C. diverg - *Carnobacterium divergens*, B. altit 1 - *Bacillus altitudinis* 1, B. altit 2 - *B. altitudinis* 2, B. pumil 1 - *Bacillus pumilus* 1, B. pumil 2 - *B. pumilus* 2, B. pumil 3 - *B. pumilus* 3; GM-gentamicin, Cl – ciprofloxacin, Tet – tetracycline, AM – amoxicillin, Amp – ampicillin, EM – erythromycin, RI – rifampicin.

Higher observed MIC values indicate that a higher antibiotic concentration was required to inhibit visible growth of a given strain under the present test conditions and therefore reflect lower phenotypic susceptibility; conversely, lower observed MIC values indicate greater susceptibility. As indicated by the findings presented in Table 4, only selected between-strain comparisons were statistically significant. For gentamicin, *C. divergens* showed a significantly higher observed MIC than *B. pumilus* 2 (12.00 vs 1.00 μg/mL, respectively; *p* ≤ 0.05), whereas the remaining strains did not differ significantly from either of these strains or from each other (Table 4). For ciprofloxacin (which was tested on the *Bacillus* strains only), *B. altitudinis* 2 showed a significantly higher observed MIC than *B. pumilus* 1 (32.00 vs 1.50 μg/mL; *p* ≤ 0.05), while the other *Bacillus* strains did not differ significantly from either strain or from each other. For tetracycline, *B. altitudinis* 1 showed a significantly higher observed MIC than *C. divergens* (12.00 vs 1.50 μg/mL; *p* ≤ 0.05), whereas *B. lactis* and *B. altitudinis* 2 did not differ significantly from either strain or from each other. For amoxicillin, *B. altitudinis* 1 showed a significantly higher observed MIC than *L. plantarum* 1 and *C. divergens* (3.00 vs 0.02 and 0.04 μg/mL, respectively; *p* ≤ 0.05). For ampicillin, *L. rhamnosus* showed a significantly higher observed MIC than *C. divergens* (2.00 vs 0.02 μg/mL; *p* ≤ 0.05), whereas the remaining strains were not significantly different. For erythromycin, *B. lactis* and *B. pumilus* 3 showed significantly higher observed MICs than *C. divergens* (6.00 and 24.00 vs 0.05 μg/mL, respectively; *p* ≤ 0.05), whereas the other strains did not differ significantly from either group. For rifampicin, *C. divergens* showed a significantly higher observed MIC than *B. pumilus* 3 (12.00 vs 2.00 μg/mL; *p* ≤ 0.05), while the remaining strains were not significantly different. Overall, the Etest® data indicate between-strain variability in observed MIC values; however, because many numerical differences did not reach statistical significance, they should be regarded as descriptive observations rather than confirmed pairwise differences.

Where both assays were available, strains with higher observed Etest® endpoints often also showed higher diffusion threshold concentrations; however, direct quantitative comparison between the two methods is not appropriate.

### 3.3 Antagonistic activity of probiotic cell-free supernatants against indicator poultry-associated and foodborne pathogens

The antagonistic activity of CFS obtained from the probiotic cultures was evaluated against four indicator poultry-associated and foodborne pathogens: *E. fergusonii*, *L. monocytogenes*, *S.* Enteritidis and *S.* Typhimurium (Fig. 3a–d). To explore the potential contribution of enzyme-sensitive inhibitory factors, CFS were additionally treated with catalase or lipase prior to pathogen exposure.

**Fig. 3:**
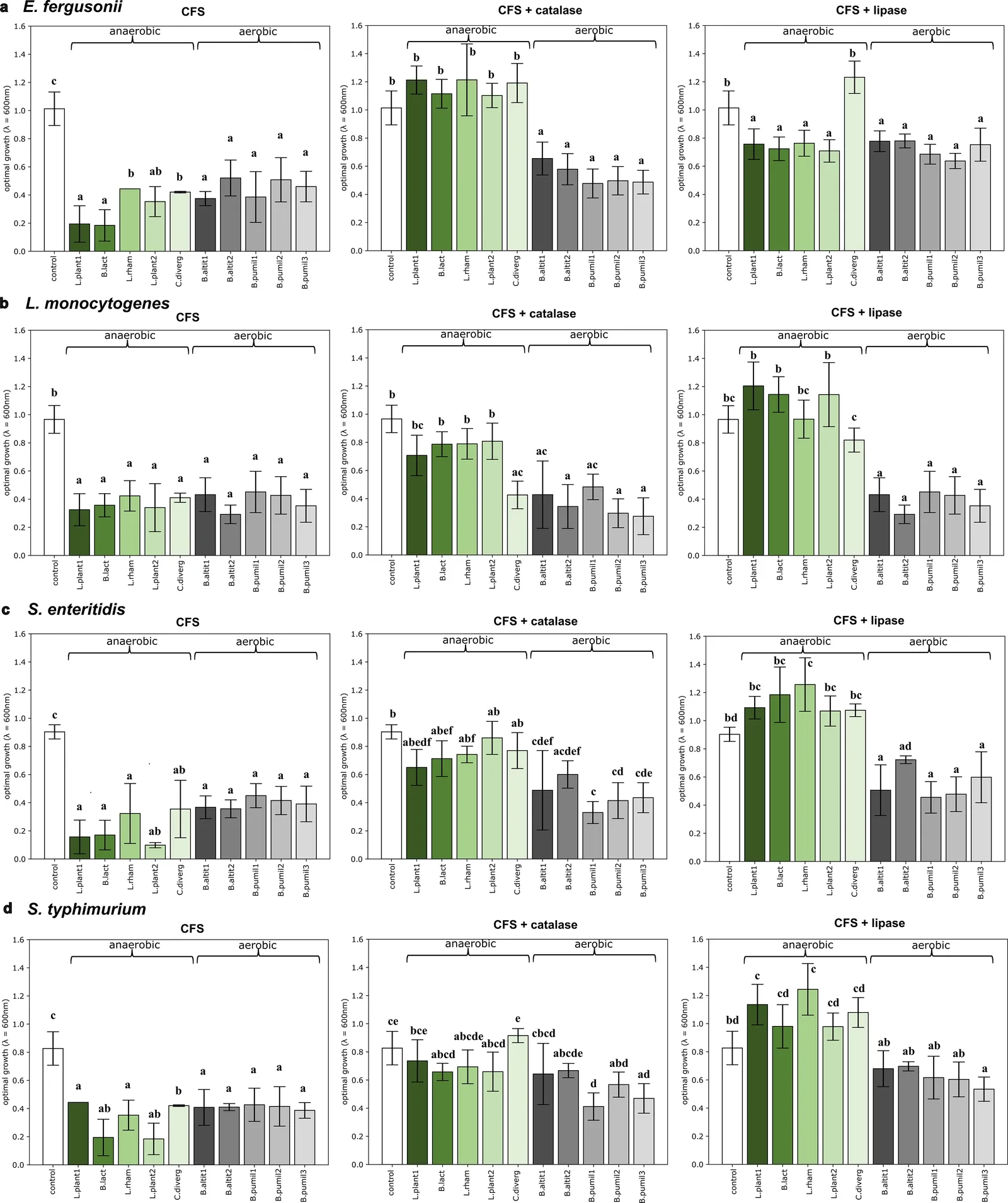
Antagonistic activity of probiotic cell-free supernatants (CFS) against selected poultry-associated and foodborne indicator pathogens: *Escherichia fergusonii* (a), *Listeria monocytogenes* (b), *Salmonella* Enteritidis (c), and *Salmonella* Typhimurium (d). ^1^Determined using a microtiter plate growth inhibition assay. For each poultry-associated and foodborne pathogen, growth in the presence of untreated CFS, catalase-treated CFS, and lipase-treated CFS was compared with pathogen growth in the control without any CFS added. Poultry-associated and foodborne pathogen growth was quantified as optical density at 600 nm (OD600) after 24 h of incubation at 37 °C. Data are presented as mean ± standard deviation (*n* = 6). Within each pathogen and within each treatment, bars that do not share a common letter are significantly different (*p* ≤ 0.05; one-way ANOVA followed by Tukey's HSD test).

For *E. fergusonii* (Fig. 3a), untreated CFS from all probiotic strains significantly reduced pathogen growth relative to the control without CFS (*p* < 0.05). Within the untreated-CFS group, OD_600_ values for *L. rhamnosus* and *C. divergens* were significantly higher than those for *L. plantarum* strain 1, *B. lactis*, *B. altitudinis* 1, *B. altitudinis* 2, *B. pumilus* 1, *B. pumilus* 2, and *B. pumilus* 3 (*p* ≤ 0.05), whereas *L*. *plantarum* strain 2 showed an intermediate value and did not differ significantly from either group. Following catalase treatment, CFS from *L. plantarum* strain 1, *B. lactis*, *L. rhamnosus*, *L. plantarum* strain 2, and *C. divergens* did not differ significantly from the control, whereas catalase-treated CFS from all *Bacillus* strains still produced significantly lower OD600 values than the control (*p* ≤ 0.05). Following lipase treatment, CFS from L. *plantarum* ATCC 11974, *B. lactis*, *L. rhamnosus*, *L. plantarum* strain 2, and all *Bacillus* strains remained significantly different from the control (*p* ≤ 0.05), whereas lipase-treated CFS from *C. divergens* did not differ significantly from the control. Thus, for *E. fergusonii*, catalase treatment abolished statistically detectable inhibition for *L. plantarum* ATCC 11974, *B. lactis*, *L. rhamnosus*, *L. plantarum* strain 2, and *C. divergens*, while lipase treatment abolished it only for *C. divergens*. For *L. monocytogenes* (Fig. 3b), untreated CFS from all probiotic strains yielded significantly lower OD600 values than the control without CFS (*p* ≤ 0.05), and no significant differences were detected among the probiotic strains within the untreated-CFS group. Following catalase treatment, CFS from *L. plantarum* ATCC 11974, *B. lactis*, *L. rhamnosus*, and *L. plantarum* strain 2 did not differ significantly from the control, whereas catalase-treated CFS from *C. divergens* and all *Bacillus* strains still gave significantly lower OD600 values than the control (*p* ≤ 0.05). Following lipase treatment, CFS from *L. plantarum* ATCC 11974, *B. lactis*, *L. rhamnosus*, *L. plantarum* strain 2, and *C. divergens* did not differ significantly from the control, whereas lipase-treated CFS from all *Bacillus* strains remained significantly lower than the control (*p* ≤ 0.05). For *S.* Enteritidis (Fig. 3c), untreated CFS from all probiotic strains yielded significantly lower OD_600_ values than the control without CFS (*p* ≤ 0.05), and no significant differences were detected among the probiotic strains within the untreated-CFS group. Following catalase treatment, CFS from ^L.^ *plantarum* ATCC 11974, *B. lactis*, *L. rhamnosus*, *L. plantarum* strain 2, and *C. divergens* did not differ significantly from the control, whereas catalase-treated CFS from all *Bacillus* strains still gave significantly lower OD_600_ values than the control (*p* ≤ 0.05). Following lipase treatment, CFS from *L. plantarum* ATCC 11974, *B. lactis*, *L. plantarum* strain 2, *C. divergens*, and *B. altitudinis* 1 or 2 did not differ significantly from the control; lipase-treated CFS from *L. rhamnosus* showed a significantly higher OD600 value than the control; and lipase-treated CFS from *B. altitudinis* 1, *B. pumilus* 1, *B. pumilus* 2, and *B. pumilus* 3 showed significantly lower OD600 values than the control (*p* ≤ 0.05). For *Salmonella* Typhimurium (Fig. 3d), untreated CFS from all probiotic strains yielded significantly lower OD600 values than the control without CFS (*p* ≤ 0.05). Within the untreated-CFS group, OD600 for *C. divergens* was significantly higher than for *L. plantarum* ATCC 11974, *L. rhamnosus*, *B. altitudinis* 1, *B. altitudinis* 2, *B. pumilus* 1, *B. pumilus* 2, and *B. pumilus* 3, whereas *B. lactis* and *L. plantarum* strain 2 showed intermediate values and did not differ significantly from either group. Following catalase treatment, CFS from *L. plantarum* ATCC 11974, *B. lactis*, *L. rhamnosus*, *L. plantarum* strain 2, *C. divergens*, *B. altitudinis* 1, and *B. altitudinis* 2 did not differ significantly from the control, whereas catalase-treated CFS from *B. pumilus* 1, *B. pumilus* 2, and *B. pumilus* 3 still gave significantly lower OD600 values than the control (*p* ≤ 0.05). Following lipase treatment, CFS from *B. lactis*, *L. plantarum* strain 2, *C. divergens*, *B. altitudinis* 1, *B. altitudinis* 2, *B. pumilus* 1, and *B. pumilus* 2 did not differ significantly from the control; lipase-treated CFS from *L. plantarum* ATCC 11974 and L. *rhamnosus* showed significantly higher OD600 values than the control; and lipase-treated CFS from *B. pumilus* 3 showed a significantly lower OD600 value than the control (*p* ≤ 0.05).

Overall, CFSs derived from all strains exhibited strong antagonistic activity against all of the tested pathogens. Enzyme-based treatments indicate that catalase-sensitive and lipase-sensitive factors contribute to the activity of the anaerobic/facultatively anaerobic strains. However, this was not the case for the *Bacillus* strains, except for the anti-*S.* Typhimurium activity of the *B. altitudinis* strains.

## 4 Discussion

### 4.1 Antibiotic use in poultry production and the One Health perspective

The extensive use of antibiotics in poultry production is widely recognized as a major driver of AMR, with consequences extending beyond animal health to humans and the environment [[98], [99]]. Antibiotic residues disseminated through manure and wastewater contribute to environmental contamination and facilitate the spread of antibiotic resistance genes (ARG) across ecosystems [100]. High concentrations of antibiotic residues and ARG, when applied to soil, can be transferred to crops, water bodies, and sediments, result in the cycling and persistence of MDR bacteria and ARG in agricultural environments [101]. Probiotic strains, including *Lactobacillus* and *Bifidobacterium*, have been shown to harbor transferable ARG, such as tetW, which can be mobilized via genetic elements and potentially disseminate resistance within the gut and the environment [102]. These interconnected risks underscore the necessity of a One Health approach that integrates animal, human, and environmental health when evaluating promising candidates for further evaluation as part of antibiotic-reduction strategies in poultry farming [103]. Within a One Health framework, antimicrobial use in poultry exerts selection pressure that can promote AMR within animal-associated microbiota, which may then be disseminated via manure, wastewater, and dust into the environment, enter the food chain through contaminated products, and ultimately contribute to human exposure. Recent EFSA/ECDC reports continue to frame AMR surveillance across humans, animals, and food within this integrated One Health system, reinforcing the relevance of our findings to broader AMR mitigation strategies. The in vitro profiling performed in the current study supports the importance of including antibiotic susceptibility screening as one component of candidate probiotic prioritization, in line with regulatory expectations for microorganisms intended for food/feed-chain use.

### 4.2 Antibiotic susceptibility profiles of probiotic candidates: implications for safety

A critical requirement for the safe application of probiotics in food and feed systems is a thorough evaluation of their antibiotic susceptibility profiles [104]. In the present study, Etest®-based MIC determination revealed pronounced strain- and antibiotic-specific variability among the tested probiotic candidates. Such heterogeneity highlights the importance of strain-level assessment rather than species-level generalization, which has also been emphasized in previous studies on probiotic safety and efficacy [99]. For example, in other studies, some strains of *Bifidobacterium longum* and *B. animalis* subsp. *lactis* exhibited high MICs to tetracyclines or streptomycin, while others remained susceptible, and similar heterogeneity was observed among *Lactobacillus* and *Pediococcus* strains [105]. A similar strain-dependent heterogeneity was observed here, particularly for erythromycin and gentamicin across the tested candidates. Elevated gentamicin endpoints in the lactic acid bacteria are notable, because reduced aminoglycoside susceptibility has previously been reported in *Lactobacillaceae* and may reflect intrinsic physiological traits rather than acquired, mobile resistance determinants.

The diffusion-based antibiotic screening provided complementary insight into comparative inhibition patterns across a wider range of antibiotics. As this approach does not allow categorical resistance interpretation in the absence of validated breakpoints, it should be regarded as a preliminary screening tool rather than definitive susceptibility testing, consistent with recommendations for probiotic evaluation [106]. The observed elevated MICs in some *Bacillus* isolates may reflect intrinsic or species-associated reduced susceptibility, but genomic confirmation would be required before any inference can be made regarding the underlying determinants or their mobility. For example, the *Bacillus* strains exhibited high MICs for ciprofloxacin and tetracycline, albeit a full comparison is not available for the *Lactobacillus* and *Bifidobacterium* strains in the current study. One *B. pumilus* strain also showed a high MIC for erythromycin. However, it should be noted that the *Bacillus* strains evaluated in the present study were previously shown to be sensitive to the panel of nine antibiotics against which EFSA recommends that *Bacillus* strains are tested, including tetracycline and erythromycin, with MIC values determined to be below EFSA breakpoints by a validated broth microdilution assay [26]. The elevated Etest® endpoints observed here for certain antibiotics therefore likely reflect methodological differences rather than confirmed resistance of regulatory concern. Elevated MICs in *Bacillus* species often reflect species-specific, chromosomally encoded resistance genes, such as those for tetracycline efflux (tetL) or erythromycin resistance (ermD, ermK), which are taxonomically conserved and do not pose a significant risk for horizontal gene transfer to pathogenic bacteria [107]. From an EFSA perspective, the key safety concern is not intrinsic antibiotic resistance per se, but the presence of acquired AMR genes relevant to human or veterinary therapy and their potential mobility. Phenotypic susceptibility testing alone cannot determine whether an observed resistance phenotype is intrinsic, chromosomally encoded, acquired, or potentially transferable. Within the QPS framework, absence of acquired genes conferring resistance to therapeutic antimicrobials is a generic qualification for bacterial taxa, while *Bacillus* strains additionally require demonstration of lack of toxigenic potential. Since whole-genome analysis was not performed here and susceptibility testing was not designed as a full regulatory package, the present data should be interpreted as preliminary strain screening information that can support candidate selection, but not as a stand-alone regulatory safety conclusion. Furthermore, comparisons of MIC values between bacterial taxa should be made with caution because observed MIC values may reflect not only intrinsic susceptibility but also differences in growth rate, medium composition, incubation atmosphere, incubation temperature, and physiological state. Statistical comparisons presented here are thus exploratory and are meant to highlight descriptive observations rather than definitive susceptibility rankings across taxa. Future studies will include whole-genome sequencing of the prioritized strains to identify (1) acquired AMR determinants, distinguish them from natural or chromosomally encoded characteristics, and evaluate their potential mobility; and (2) virulence-associated and toxin genes in accordance with EFSA guidance on microorganism characterization in the food/feed chain.

### 4.3 Antagonistic activity of probiotics and influence of cultivation conditions

Probiotic antagonism against pathogenic bacteria is commonly attributed to the production of compounds with antimicrobial activity, including organic acids, hydrogen peroxide, bacteriocins, and other bioactive metabolites [[108], [109]]. In the present study, CFS from all the test strains inhibited the growth of all tested poultry-associated and foodborne pathogens, both Gram-negative (*E. fergusonii*, *S.* Enteritidis, and *S.* Typhimurium) and Gram-positive (*L. monocytogenes*), with some differences observed depending on the probiotic strain (Fig. 3). The antagonism experiment exceeded the conventional CLSI/EUCAST broth microdilution inoculum recommendations by using a higher than recommended pathogen inoculum per microtitre plate well.

The *Bacillus* strains included in the present panel had previously been characterized for antimicrobial activity in studies by Prieto et al. [25]. In that work, the corresponding seaweed-derived *Bacillus* isolates showed anti-listerial activity in CFS when tested by agar well diffusion assay, and activity against *Staphylococcus aureus* was also reported [25]. Importantly, Prieto et al. [26] also showed that activity against Gram-negative indicators, including *S.* Typhimurium, was not detected in the CFS but was observed in deferred antagonism assays with growing cultures; among the *Bacillus* strains used here, this anti-*Salmonella* activity was reported for all except the *Fucus serratus*-derived *B. altitudinis* strain [25], [26] Therefore, the present microtiter CFS assay extends these earlier well-diffusion and deferred-antagonism observations to poultry-associated pathogens and highlights that the detectable antimicrobial spectrum of *Bacillus*-derived activity may depend strongly on the assay format, growth conditions, and whether CFS or growing cultures are tested. Enzyme-based treatments with catalase and lipase produced strain- and pathogen-dependent changes in detectable inhibitory activity. The loss of detectable activity following catalase treatment is consistent with a contribution of hydrogen peroxide or other catalase-sensitive factors, but does not prove hydrogen peroxide as the sole causal compound. Similarly, reductions in antimicrobial activity after lipase treatment suggest a possible contribution of lipase-sensitive or lipid-associated factors in selected strain–pathogen combinations, but do not identify a specific lipid-mediated antimicrobial mechanism. Hydrogen peroxide-mediated inhibition has frequently been reported for *Lactobacillus* strains, particularly against *Salmonella* spp. and *E. coli* [108]. It should be noted that the *B. lactis* strain tested in the current study was cultivated under CO₂-enriched conditions (5% CO₂), which do not correspond to strict anaerobiosis. Previous research suggests that bifidobacteria may show slower growth and altered metabolic profiles under microaerophilic or CO₂-enriched conditions, including shifts in the lactate-to-acetate ratio due to changes in fermentative pathways. However, detailed growth-kinetics comparisons with strict anaerobic conditions were not carried out in the current study. The composition and efficacy of CFS may be impacted by these metabolic variations. To measure such effects, additional research comparing strictly anaerobic and CO₂-enriched incubation conditions for these strains would be beneficial.

In other mechanistic studies, Muñoz-Quezada et al. [110] demonstrated that *Lactobacillus paracasei*, *Bifidobacterium breve* and *L*. *rhamnosus* isolated from the faeces of breast milk-fed infants competitively inhibited the pathogens tested (*S.* Typhimurium and enterotoxigenic *E. coli*) through the production of antimicrobial compounds in their CFS. The inhibitory effect was strain-specific and dependent on cultivation conditions, with some supernatants maintaining activity after neutralization and others losing it, indicating that organic acids and possibly bacteriocins or other metabolites contributed to the antagonism [110]. However, in contrast to studies that explicitly demonstrated acid-mediated inhibition through pH-controlled experiments [111], the present study did not include pH-neutralization controls. Therefore, while organic acids are well-established contributors to probiotic antagonism, their specific role in the observed inhibitory effects cannot be confirmed or excluded here. Furthermore, because protease treatment was not performed, the present data do not allow conclusions regarding bacteriocins or other proteinaceous inhibitors. For the *Bacillus*-derived CFS, the limited impact of catalase and lipase in most comparisons does not exclude the involvement of other inhibitory compounds, including proteinaceous factors. This possibility is supported by the previous physicochemical characterization reported by Prieto et al. [25], in which CFS-associated antimicrobial activity of marine *Bacillus* isolates was sensitive to several proteolytic enzymes. However, because protease treatment was not performed in the present study, proteinaceous or bacteriocin-like activity should be regarded as a hypothesis derived from previous literature rather than a conclusion supported by the current experiment.

### 4.4 Relevance of probiotic antagonism as an antibiotic-reduction strategy

Probiotics with demonstrated antagonistic activity against poultry and food safety-associated pathogens represent promising candidates for reducing antibiotic use in animal production [[112], [113]]. By suppressing pathogen growth and supporting gut microbial balance, probiotics may help to mitigate infection pressure and improve animal health and productivity [114]. Previous studies have highlighted the potential of *Bacillus*-based probiotics to control *Salmonella* spp. through antagonistic activity and interference with biofilm formation [115]. *Bacillus*-based probiotics and *L*. *rhamnosus* have demonstrated efficacy in controlling foodborne pathogens in poultry, particularly *Salmonella* spp.*, Campylobacter* spp.*, Clostridium perfringens,* and *E. coli* [[116], [117], [118]]*.* In vitro, a *Bacillus safensis* CFS showed dose-dependent inhibition of multiple *Campylobacter jejuni* strains and based on assessment in BALB/c mice was demonstrated safe, supporting its potential as a probiotic candidate, both for poultry health management and food safety improvement [119]. By suppressing foodborne and poultry-associated pathogens, selected probiotic candidates may represent promising candidates for further genomic, mechanistic, and in vivo evaluation and antibiotic-reduction strategies in poultry farming.

Nevertheless, the translation of in vitro antagonistic effects demonstrated in this study into practical applications requires careful consideration of antibiotic susceptibility, safety, and regulatory compliance. The present findings therefore provide an important screening step that supports the selection of promising probiotic candidates while emphasizing the need for further validation.

### 4.5 Study limitations and future perspectives

The antagonistic activity towards poultry- and food safety-associated pathogens observed in vitro supports further evaluation of selected candidate strains as part of broader antibiotic-reduction strategies in poultry production. Previous studies have suggested that probiotic- and microbiota-based interventions may contribute to pathogen control and intestinal health, but their effectiveness depends strongly on strain-specific properties, host context, diet, management conditions, and validation in vivo. Therefore, the present results should be interpreted as an initial screening outcome that identifies candidate probiotic strains for further genomic, mechanistic, and poultry-based validation rather than as evidence of demonstrated antibiotic-use reduction or improved food safety. Future studies should focus on genomic profiling and in vivo efficacy trials. By prioritising the most promising probiotic strains and excluding candidates with limited activity, the in vitro results provide a scientific basis for subsequent investigations and appropriately designed poultry trials, while reducing unnecessary animal use and avoiding poorly justified experimental treatments.

## 5 Conclusions

The present study identified strain-dependent differences in observed antibiotic-response profiles and CFS-based antagonistic activity among candidate probiotic strains relevant to poultry production. The combined use of qualitative disk-diffusion screening, Etest® MIC determination, and in vitro pathogen-growth inhibition assays provides a cautious framework for prioritising candidates for further study. The antagonistic effects observed here are consistent with multifactorial inhibitory activity, but their mechanisms require additional confirmation. Overall, the findings support the selection of candidate strains for further genomic, mechanistic, and in vivo validation rather than providing stand-alone evidence of probiotic efficacy, regulatory safety, or food-safety improvement.

## CRediT authorship contribution statement

**Niloofar Akhavan:** Writing – original draft, Investigation, Formal analysis. **Katarzyna Stadnicka:** Writing – review & editing, Writing – original draft, Validation, Supervision, Resources, Project administration, Methodology, Investigation, Funding acquisition, Formal analysis, Conceptualization. **Dominika Thiem:** Writing – review & editing, Visualization, Validation, Software, Methodology, Formal analysis, Data curation. **Gillian E. Gardiner:** Writing – review & editing, Resources. **Peadar G. Lawlor:** Writing – review & editing, Resources. **Cinzia Lucia Randazzo:** Writing – review & editing, Resources. **Katarzyna Hrynkiewicz:** Writing – review & editing, Writing – original draft, Visualization, Validation, Supervision, Resources, Methodology, Investigation, Formal analysis, Conceptualization.

## Ethics approval

Not applicable. This study involved only in vitro experiments using bacterial strains and did not involve human participants, human material, live animals, animal procedures, or samples requiring ethical approval.

## Funding

The research is funded by The National Science Centre in Poland, UMO-2019/35/B/NZ9/03186 OVOBIOM.

## Declaration of competing interest

The authors declare that the research was conducted in the absence of any commercial or financial relationships that could be construed as a potential conflict of interest.

## Data Availability

All data generated and analysed during this study are included in this published article and its supplementary information files. Additional data supporting the findings of this study are available from the corresponding author upon reasonable request.
